# Supplementary material for: Coordination of parental performance is breeding phase-dependent in the Dovekie (Alle alle), a pelagic Arctic seabird
Source: PLoS One. 2024 Sep 4;19(9):e0306796. doi: 10.1371/journal.pone.0306796 (PMC11373810; doi:10.1371/journal.pone.0306796)
Supplement: S1 File — Containing detailed information on egg development phases and age of the chick during recording sessions, on the threshold used for short trip/ long trip classification, as well as on the bootstrap procedure used to evaluate the estimates of the models. (DOCX) [file pone.0306796.s001.docx]

**Coordination of parental performance is breeding phase-dependent in the Dovekie (*Alle alle*), a pelagic Arctic seabird**

Running title: *Changes in parental coordination during breeding*

**Supplementary Information**

**Table S1. Egg development phases and age of the chick.** Values are expressed in number of days before (resp. after hatching date), at the beginning of the recording session.

| **Recording Session** | **Year** | **Mean age (in days)** | **Min age (in days)** | **Max age (in days)** |
| --- | --- | --- | --- | --- |
| early incubation | 2019 | 26 | 24 | 27 |
|  | 2020 | 20 | 16 | 27 |
|  | combined | 23 | 16 | 27 |
| mid incubation | 2019 | 15 | 13 | 17 |
|  | 2020 | 12 | 8 | 19 |
|  | combined | 14 | 8 | 19 |
| late incubation | 2019 | 5 | 4 | 7 |
|  | 2020 | 4 | 2 | 11 |
|  | combined | 5 | 2 | 11 |
| early chick rearing | 2019 | 3 | 3 | 4 |
|  | 2020 | 4 | 4 | 5 |
|  | combined | 4 | 3 | 5 |
| mid chick rearing | 2019 | 11 | 11 | 12 |
|  | 2020 | 12 | 12 | 12 |
|  | combined | 11 | 11 | 12 |

**Table S2. Threshold used for the classification of short and long trips during th chick rearing period.** Values are expressed in number of hours.

| **Recording Session** | **Year** | **Threshold (in hours)** | **Mean duration of Short Trips (in hours)** | **Mean duration of Long Trips (in hours)** |
| --- | --- | --- | --- | --- |
| early chick rearing | 2019 | 6.10 | 1.77 | 11.41 |
|  | 2020 | 6.00 | 2.20 | 11.61 |
| mid chick rearing | 2019 | 8.85 | 1.9 | 17.12 |
|  | 2020 | 8.55 | 2.30 | 16.14 |

**Link between the coordination in incubation and chick rearing - models evaluation**

**# early chick rearing**

To test the link between parental coordination during early chick rearing period and particular stages of the incubation period, linear model was applied (main text), with coordination index at early chick rearing as a response variable and coordination at three stages of the incubation as fixed effects. The model had relatively good fit (Fig. S1) and explained 31% of variance (R = 0.31).


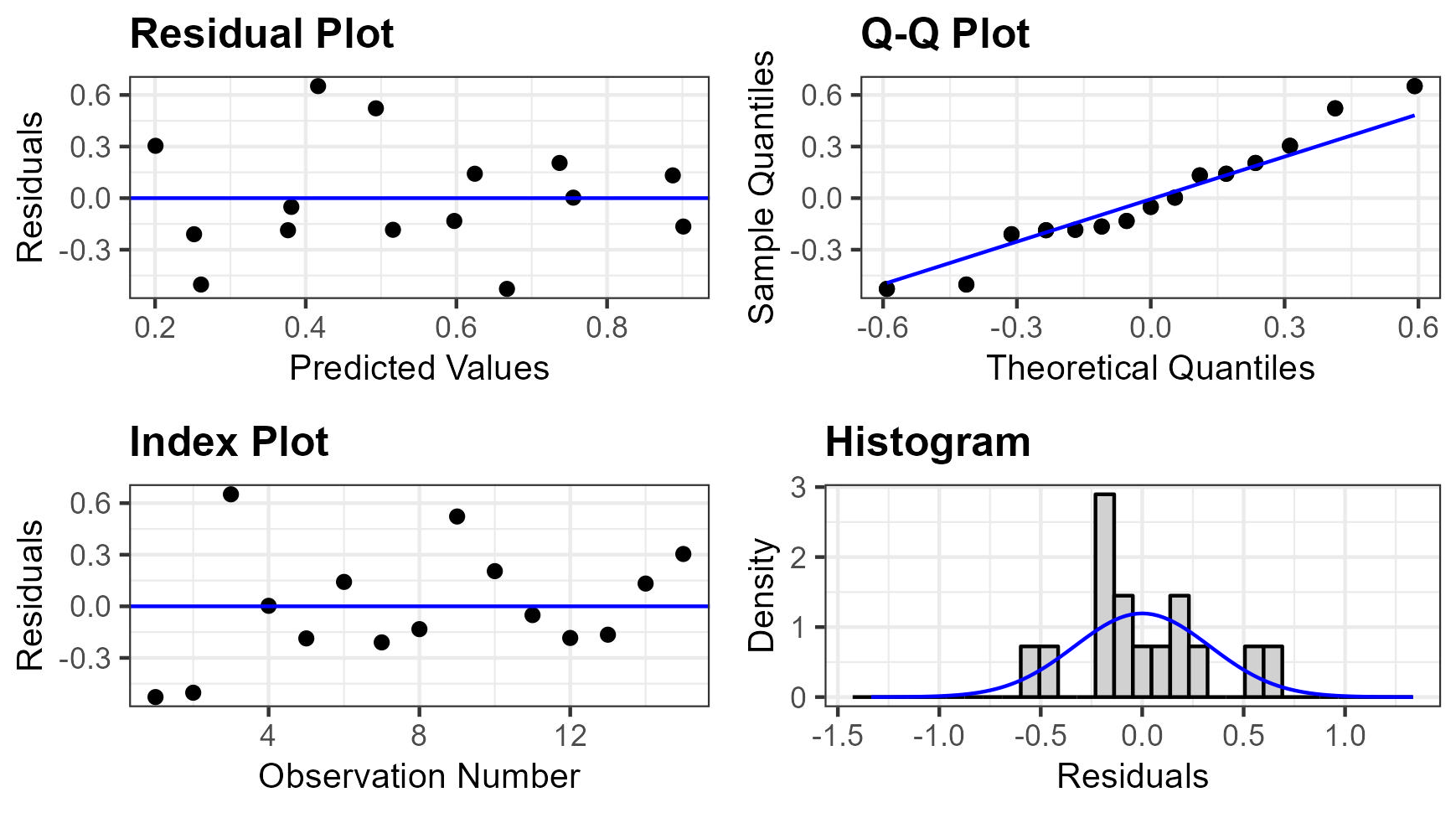


**Figure S1.** Diagnostic plots for the model testing the link between parental coordination at

**early chick rearing** period and particular stages of the incubation period.

To analyse data points for possible outliers, Cook’s distance was considered (Fig. S2). One or two points might be outliers, due to three reasons (following Fox 1991):

1. the maximum value of Cook’s distance for one of the points is 1.07, with the threshold being 1;
2. one of the points was beyond the range of three times of the mean Cook’s distance;
3. two **values of** Cook’s distance **seemed to be substantially larger than the rest**.


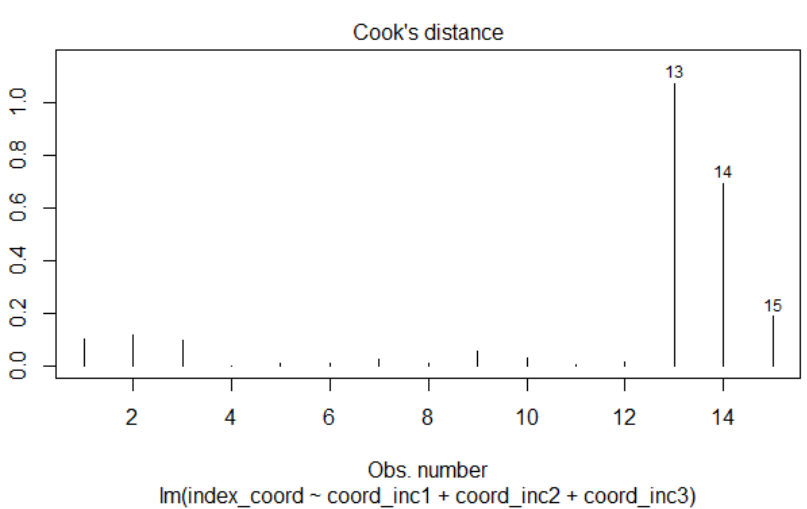


**Figure S2.** Cook’s distance for all the data points in the model on the link between parental coordination index during the **early chick rearing period** and coordination during the incubation stages.

Due to possible outliers and or influential points, the estimates of the model on the link between parental coordination index during the **early chick rearing period** and coordination during the incubation stages were tested with a bootstrap procedure. To this end, the data points were sampled with replacement and the model was run with randomized values. The procedure was repeated 10 000 times. The bootstrap estimates were then plotted and their distribution was analysed in respect to 0. P-value of each estimate was calculated as the proportion of cases with the estimate value being lower or higher than 0 (depending on the sign of the estimate from the model obtained in the standard approach).

Compared to the model output obtain with a standard approach, p-value for early and late incubation remains negative but becomes significant, while for mid incubation it remains insignificant (Fig. S3).


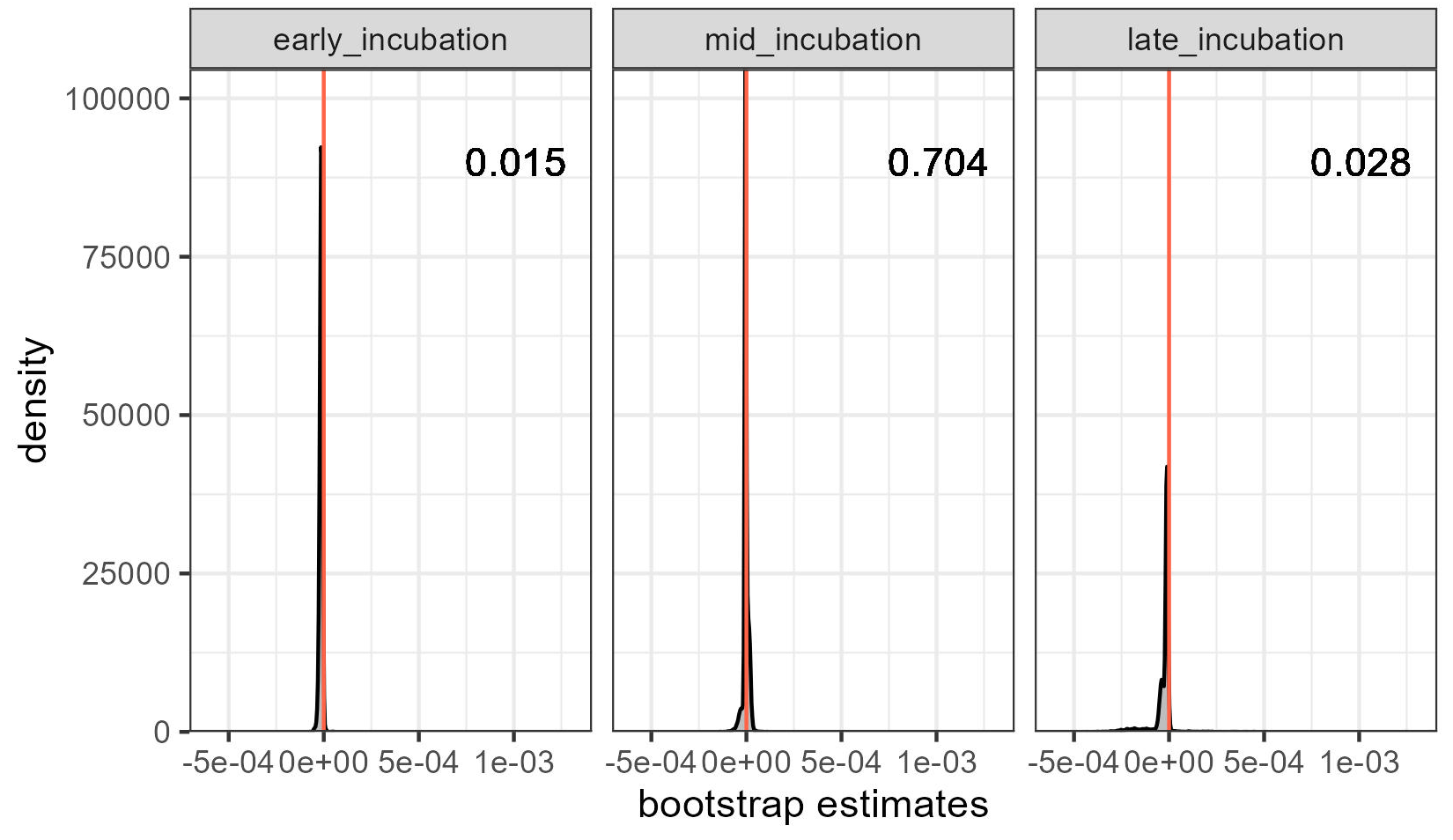


**Figure S3.** Distribution of the bootstrap estimates of the model on the link between parental coordination index during the **early chick rearing period** and coordination during the incubation stages. P-values for the estimates are denoted in the upper right corner of each panel.

**# mid chick rearing**

To test the link between parental coordination during mid chick rearing period and particular

stages of the incubation period, linear model was applied (main text), with coordination index

at mid chick rearing as a response variable and coordination at three stages of the incubation

as fixed effects. The model had relatively good fit (Fig. S4) and explained 62% of variance

(R = 0.62).


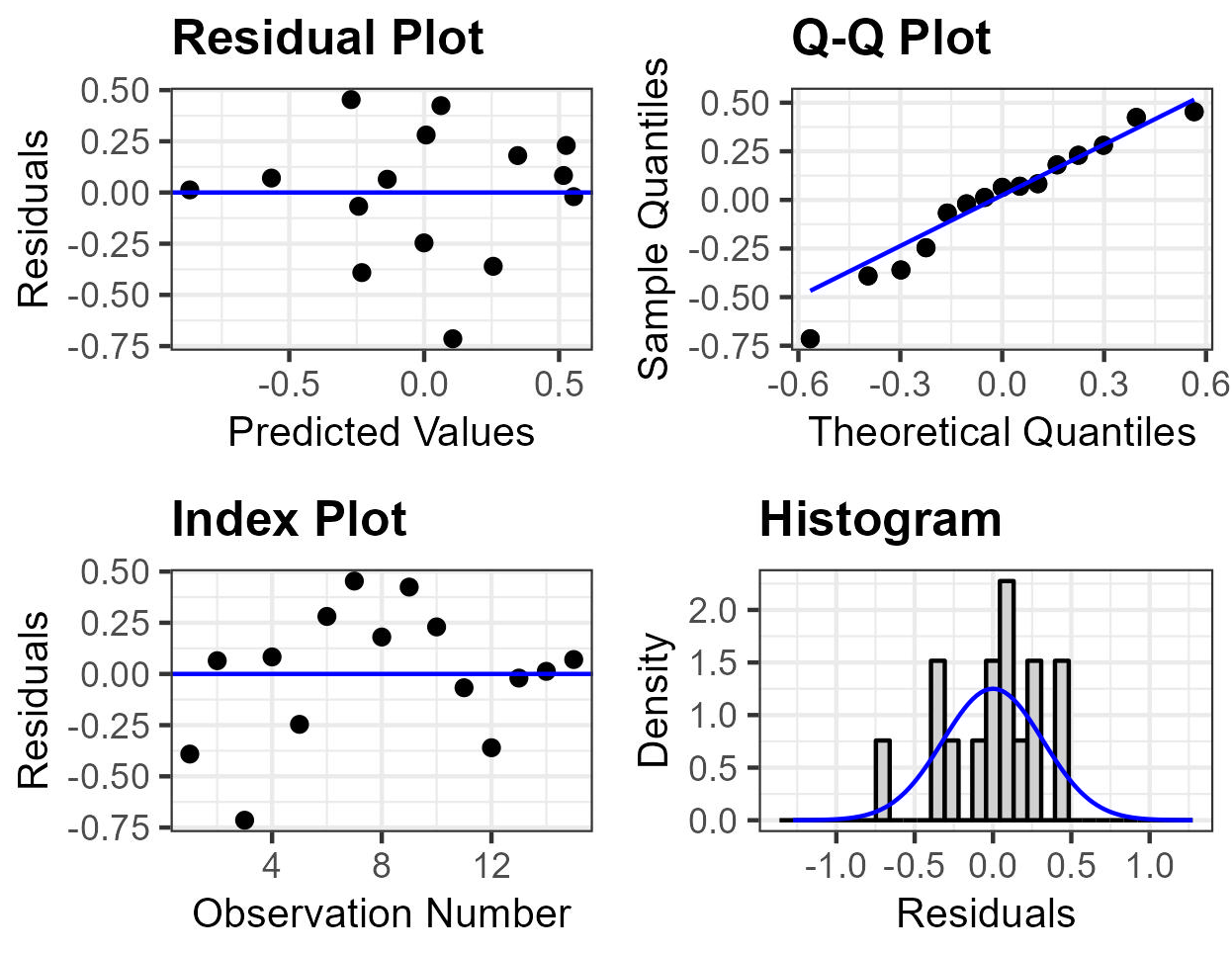


**Figure S4.** Diagnostic plots for the model testing the link between parental coordination at

**mid chick rearing** period and particular stages of the incubation period.

To analyse data points for possible outliers, Cook’s distance was considered (Fig. S5). None

of the points seems to be outlier, due to three reasons (following Fox 1991):

1. the maximum value of Cook’s distance within examined data points is 0.13 (i.e. lower than 1, which could be considered as a threshold);
2. the values of all the points were within range of three times of the mean Cook’s distance;
3. none of the **values of** Cook’s distance **were substantially larger than the rest.**


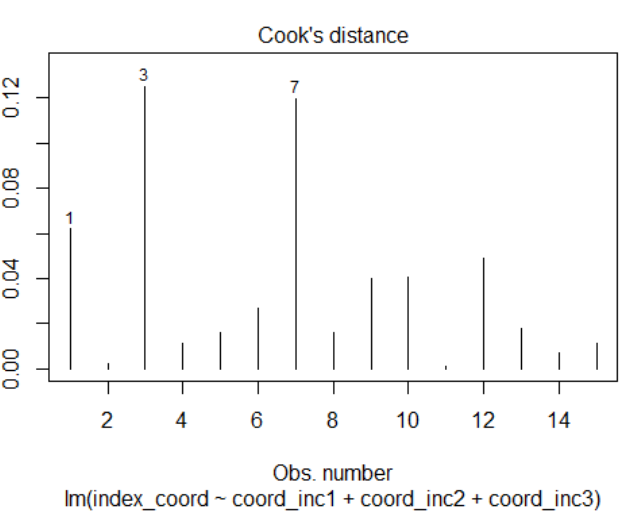


**Figure S5.** Cook’s distance for all the data points in the model on the link between parental coordination index during the **mid chick rearing period** and coordination during the incubation stages. None of the points

Although there was no apparent outliers, for consistency with the analysis for the early chick rearing period and relatively small sample size, the estimates of the model on the link between parental coordination index during the **mid chick rearing period** and coordination during the incubation stages were tested with a bootstrap procedure. To this end and as for the early chick rearing stage, the data points were sampled with replacement and the model was run with randomized values. The procedure was repeated 10 000 times. The bootstrap estimates were then plotted and their distribution was analysed in respect to 0. P-value of each estimated was calculated as the proportion of cases with the estimate value being lower or higher than 0 (depending on the sign of the estimated from the model obtained in the standard approach).

Compared to the model output obtain with a standard approach, p-value for early incubation remains significant, while for mid chick rearing becomes marginally significant (92% of the estimates are higher than 0; Fig. S6). P-value for the late incubation remains insignificant (Fig. S6).


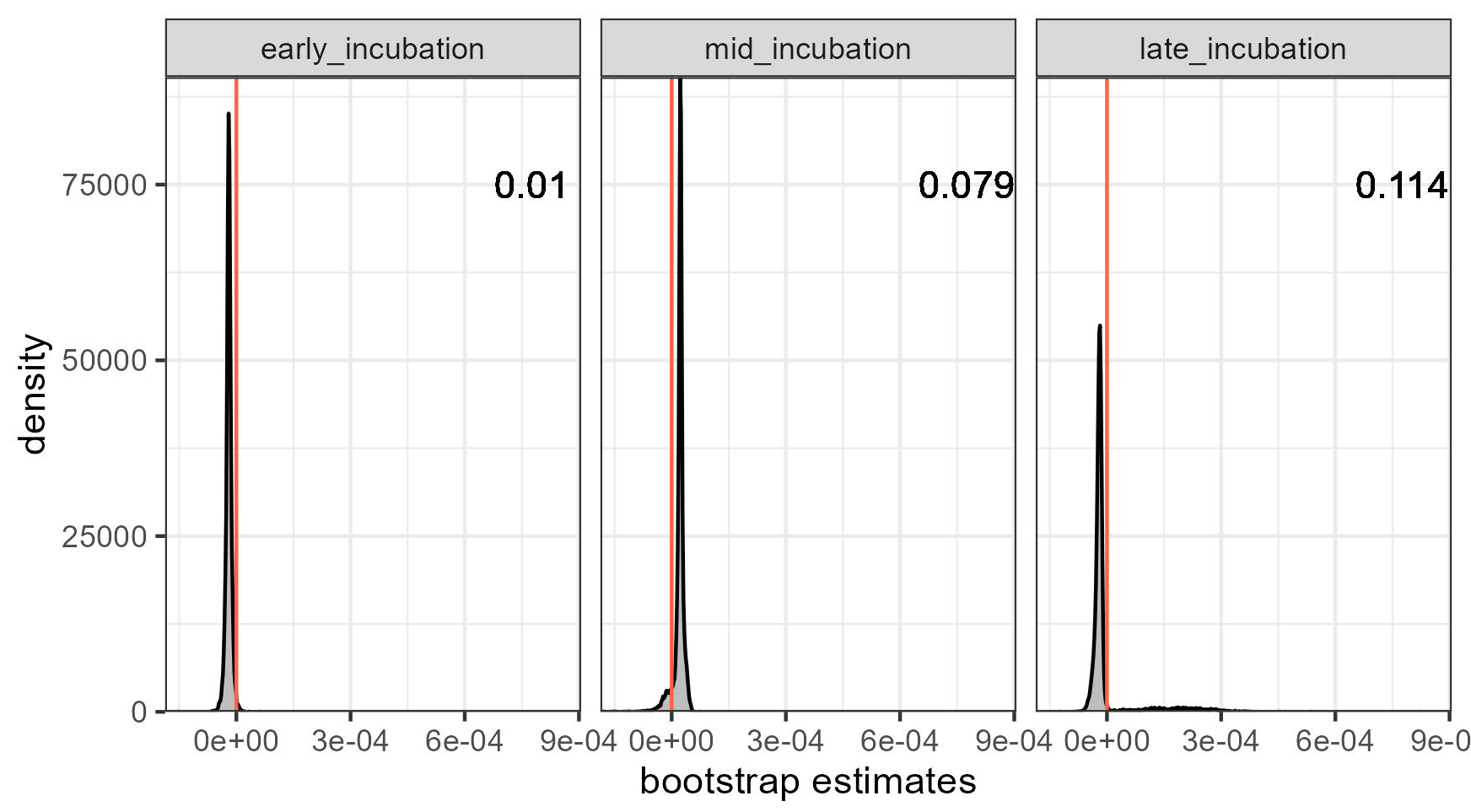


**Figure S6.** Distribution of the bootstrap estimates of the model on the link between parental coordination index during the **mid chick rearing period** and coordination during the incubation stages. P-values for the estimates are denoted in the upper right corner of each panel

**Literature cited**

Fox J. 1991. Regression Diagnostics: An Introduction. Sage Publications.
