# Supplementary figures and images for: Coordination of parental performance is breeding phase-dependent in the Dovekie (Alle alle), a pelagic Arctic seabird
Source: PLoS One. 2024 Sep 4;19(9):e0306796. doi: 10.1371/journal.pone.0306796 (PMC11373810; doi:10.1371/journal.pone.0306796)

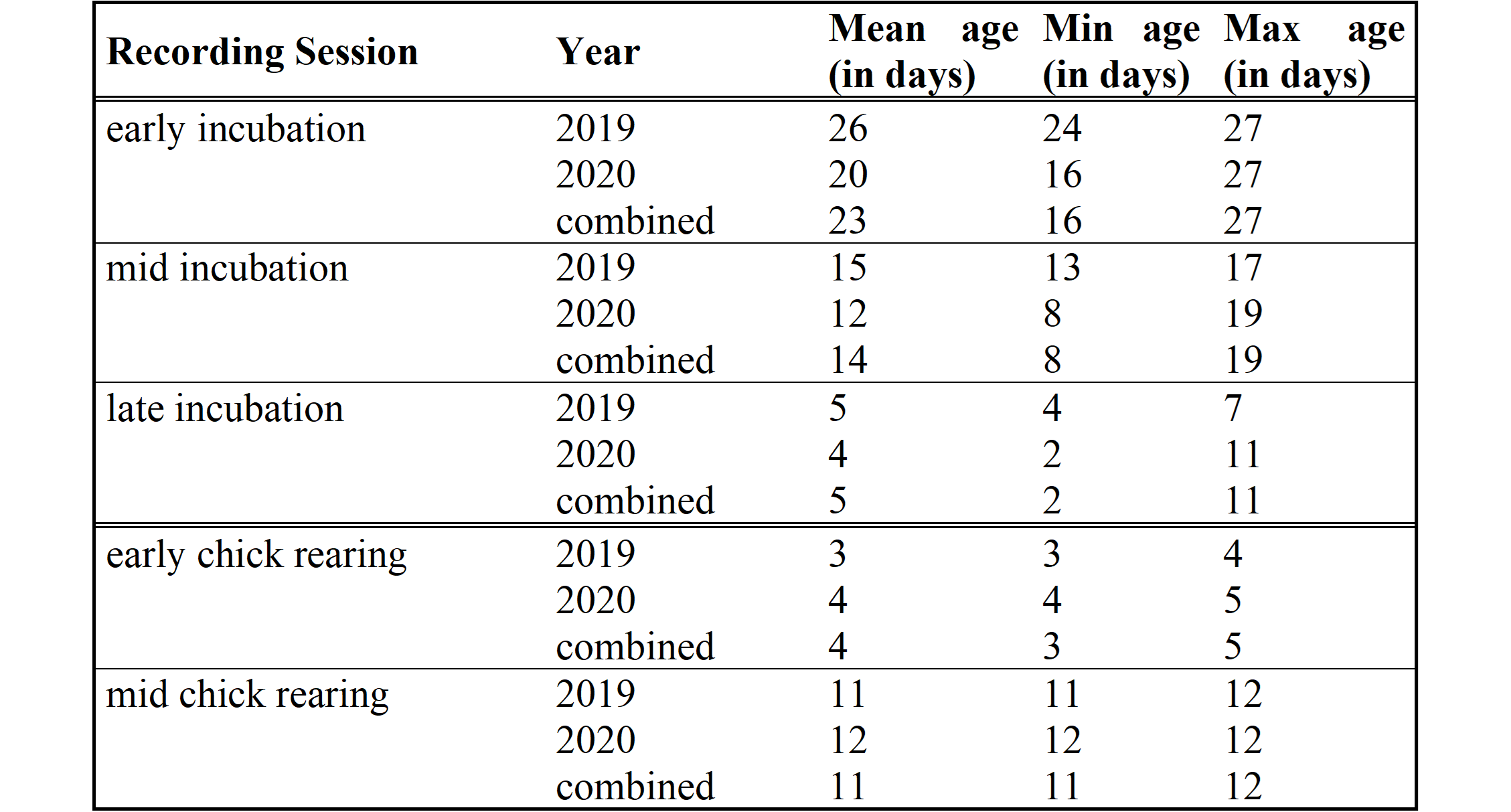

Supplement: S1 Table — Values are expressed in number of days before (resp. after hatching date), at the beginning of the recording session. (TIF) [file pone.0306796.s002.tif]

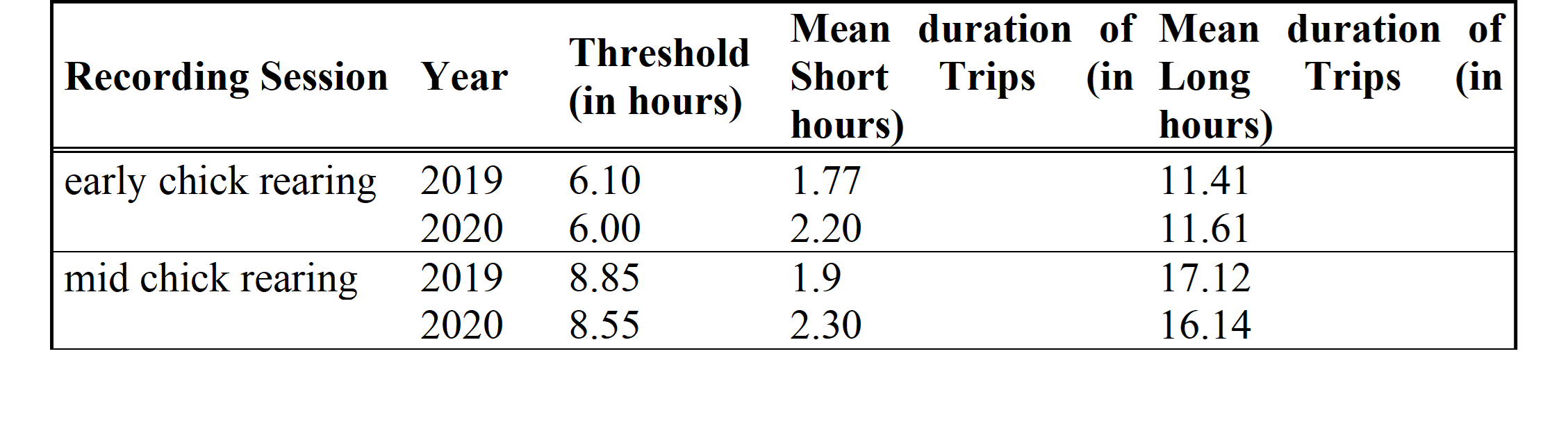

Supplement: S2 Table — Values are expressed in number of hours. (TIF) [file pone.0306796.s003.tif]
