# Supplementary material for: Coordination of parental performance is breeding phase-dependent in the Dovekie (Alle alle), a pelagic Arctic seabird
Source: PLoS One. 2024 Sep 4;19(9):e0306796. doi: 10.1371/journal.pone.0306796 (PMC11373810; doi:10.1371/journal.pone.0306796)
Supplement: Attachment — Submitted filename: PONE-D-23-40200 Done.pdf [file pone.0306796.s004.pdf]

The authors provided an interesting manuscript about "**Coordination of parental performance is breeding phase-dependent in the Dovekie (*Alle alle*), a pelagic Arctic seabird".**

| Section | Comments |
|---|---|
| **Abstract, title** | - The abstract effectively communicates the study's purpose, methods, and key findings.<br>- The language is clear and concise, making the abstract accessible to a broad audience.<br>- The rationale for the study is well-established, the study's hypotheses are implied but could be explicitly stated for added clarity and would enhance the abstract's completeness.<br>- Keywords are good, HOWEVER, it is preferable not to use the studied animal's name, as well as using a general word as Coordination, please try more specific ones. |
| **Introduction** | - The introduction is well-organized, with a logical flow from the general concept of parental care to the specific study focusing on the Dovekie and its breeding season coordination. The transition between sections is smooth. Provides a comprehensive overview of the context, research gap, and objectives. |
| **Method** | - It is well designed and could be a very helpful manual for any researcher.<br>- Line 248: please try not to use that word "manipulations" when we talk about data.<br>- Line 186 -190: need reference to confirm that it is ok to focus on successful breeders only.<br>- However, the author mentions his results in that section for more explanation. BUT it could confuse the reader as in Line 342-347. So, please it is preferable not to discuss results in that section and/or mention the tables or figures. |
| **Results Discussion** | - The data presented in an appropriate way, and contributing to the broader understanding of bi-parental care in Dovekie.<br>- The discussion contextualizes the current findings with existing literature, acknowledging previous studies on Dovekie chick.<br>- Effectively concludes by summarizing key points, emphasizing the flexibility of parental coordination, and calling for future studies to identify the exact drivers of coordination.<br>- While the study acknowledges the potential influence of environmental conditions, a more detailed examination of these factors, such as specific climatic conditions or foraging challenges, could strengthen the discussion. |
| **Overall** | - This study is well designed, and the manuscript provides valuable insights into how parental performance in a long-lived, monogamous seabird, emphasizing the flexibility of bi-parental care behavior rather than a fixed approach. It successfully navigates through the complexities of seasonal, stage-dependent, and inter-annual variations in parental coordination, contributing to the broader understanding of bi-parental care in seabirds. |
