# Supplementary material for: Coordination of parental performance is breeding phase-dependent in the Dovekie (Alle alle), a pelagic Arctic seabird
Source: PLoS One. 2024 Sep 4;19(9):e0306796. doi: 10.1371/journal.pone.0306796 (PMC11373810; doi:10.1371/journal.pone.0306796)
Supplement: Attachment — Submitted filename: Response to Reviewers.docx [file pone.0306796.s005.docx]

Dear Dr., Antoine Grissot

Thank you for submitting your manuscript to PLOS ONE. After careful consideration, we have decided that your manuscript needs Minor Revision.

Comment Editor:

It is necessary to make all amendments and corrections requested by Reviewer 2

Kind regards,

Prof. Lamiaa Mostafa Radwan, Ph.D.

Academic Editor

PLOS ONE

REPLY #1: We appreciate the editor and reviewers’ work on this manuscript and we did our best to address all the concerns left for this minor revision. Thank you for giving us this opportunity to further improve our manuscript.

Reviewer1

Accept

Reviewer2

Many thanks to the authors for their efforts in revising their manuscript. I appreciate that they have implemented some of my feedback, as well as their additional clarification regarding the coordination metrics. However, while the authors have addressed my comments in their rebuttal letter, and for the most part done so very well and clearly, I found that these changes have not consistently translated into edits to the manuscript itself. Consequently, many of my comments below are identical to those provided in the previous review, with explicit requests that the well-explained responses provided by the authors are integrated directly into the manuscript text. Rectifying this should involve little effort beyond incorporating the content from the rebuttal directly into the manuscript, as is standard practice during revisions.

REPLY #2: Thank you for your comments. We did our best to incorporate our answers from the previous rebuttal into the manuscript as requested. We also carefully proofread the whole text, further clarifying it, if necessary. Please see all the changes on the text of the manuscript.

My concerns about the analyses have been somewhat alleviated but there is one part I still do not understand – the authors make the argument that their coordination analyses work on the basis that birds can engage in three different behaviours: nest, colony, and foraging. Yet, in the rebuttal, they state:

‘Individuals when present in the colony usually spend most of their time in the surroundings of their nest. Furthermore when they leave the frame and come back with a full gular pouch, we know a foraging trip was performed. There is a possibility they spend a little time in the colony before actually departing for the foraging trip, but assume that it is negligible.’

If colony time is assumed to be negligible, I don’t understand how the rest of the analyses follow? This is very likely a misunderstanding on my part, but it has come from unclear explanations. If the authors address this, please ensure this is reflected in the manuscript rather than just the response.

REPLY #3: We agree it may be confusing, and clarified the issue. Please see the lines: 310-313. What is negligible is the time between the last appearance on the frame, and the actual departure from the colony, not the time spent in the colony. A way to correct the above statement would be “There is a possibility they spend a little time in the colony **without being seen on screen**, before actually departing for the foraging trip, but assume that it is negligible.”

Finally - this is more of a point to the editor - as the authors acknowledge, the manuscript still requires proof reading, but I note that they have made arrangements to do this for their next revision. I have therefore not addressed any typographical or grammatical errors.

REPLY #4: We carefully proofread the whole manuscript, doing our best to fix all grammatical and typographical errors. It was also proofread by Jacob Ligorria, a native English speaker and ecologist, therefore providing typographical and grammatical corrections while having a good knowledge on the ecological model and questions investigated in the study.

My relevant line-by-line comments are below, and refer to the ‘tracked changes’ version of the manuscript.

Introduction

L54: Possibly debatable whether 10 years ago is ‘recently’, I’d suggest removing this word.

REPLY #5 : We modified the text as suggested.

L61: These papers show *lower* offspring fitness due to biparental care, and so are improperly cited. The point of my original comment is that while biparental care may improve offspring fitness, this is not guaranteed. McNamara demonstrates this theoretically; Royle shows this experimentally in zebra finches. All this requires is something like ‘(*but see* Royle et al 2003, McNamara et al 2003)’ but I also suggest reading the papers as they are important pieces of theory.

REPLY #6: We are sorry for misinterpreting the original comment. We can now see the point and we rephrased the relevant text. Please see the lines: 53-63

L68: Suggest ‘reduced time investment in pair bonding’ rather than ‘short time of pair-bonding’ for clarity.

REPLY #7 : Agreed and changed, lines 75-76.

L87-88: It is still necessary to explain why environment is likely to be important i.e. give some examples of why/when environment impacts coordination and why it differs between stages. E.g. food availability might differ seasonally, which could be more important in chick rearing when energetic constraints are higher. I appreciate that the authors are investigating novel questions, but they are not plucked from thin air, it’s important to evidence the hypotheses.

REPLY #8 : We rephrased the whole two paragraph to clarify the effect of environment on the system of parental care, please see the lines: 98-133and 144-152. The fragment about an effect of the environment on the coordination at particular stages of breeding remains to be investigated, and so we are here hypothesizing differences in coordination level between the breeding stages. We would not like to elaborate the issue on environmental effect as this could mislead the readers about the direction of the paper (we are not really considering the effect of environment, we just expect that given such an effect we can expect some differences in the level of parental coordination).

L90: Similarly, there are examples in the literature that could be provided here. Even if other authors haven’t specifically compared breeding phases, there are plenty of other studies that could be used to support this e.g. those showing differences between breeding phases in trip duration, in parental behaviour, in specific constraints. ‘May thus be specific…’ is too vague.

REPLY #9: We rephrased the whole paragraph. Please see the response #8.

L96: This is the sunk cost fallacy – animals never make decisions based on prior investment alone. If a breeding attempt is doomed to fail parents will end the attempt regardless of how much prior investment they have made. Rather, to make this argument one could discuss that it is more costly to start afresh than to continue. But this is a subtle difference. Please see Dawkins & Carlisle’s original explanation: DAWKINS, R., CARLISLE, T. Parental investment, mate desertion and a fallacy. Nature 262, 131–133 (1976). https://doi.org/10.1038/262131a0

This sentence should therefore be removed. I actually think your next few sentences (discussing the idea that many birds don’t coordinate incubation but do coordinate chick rearing) illustrates the idea that coordination might be more pronounced during chick rearing just fine. You could also discuss the increased energetic costs of chick rearing vs incubation and the increased constraints associated with dividing self-care and chick care, for which the literature is full of examples.

REPLY #10: Yes, we agree that the way we phrased it sounds like the sunk cost fallacy, a topic we do not really want to explore here. We rephrased.

L122-123: It’s not enough to just discuss ‘patterns’ – please explain what you mean and give examples (e.g. lots of seabirds where foraging trips are longer during incubation than chick-rearing). As pointed out in the rebuttal, PLOS is a broad journal, so it is important to explain what this means behaviourally.

REPLY #11: We provided an example supporting it with relevant literature, see line 156.

L125: ‘Runway’ is not a term I have encountered in the literature, and I doubt I’m alone – I suggest explaining as it was in the rebuttal letter i.e. parents may need time to synchronise to each other’s behaviour prior to breeding.

REPLY #12 : Change has been made, lines 159-161.

L126: Given there is no evidence (based on the rebuttal letter) I suggest removing; ‘familiarity’ is poorly defined in the literature anyway.

REPLY #13: Removed

L157: It is essential to state that eggs can withstand neglect, and how long for. If eggs could only survive 10 minutes of neglect, then my original point that successful breeders are constrained to apparently coordinate would still stand. This is crucial to the rest of the analyses so needs to be stated explicitly and clearly.

REPLY #14: We added some information on egg neglect based on personal observations, see lines 192-196.

L166: I still don’t understand what is meant by ‘habituation to the parental mode’ even with the rebuttal letter. This needs explanation – perhaps ‘increased within-pair synchronicity’?

REPLY #15: We removed, see lines 204-205.

L244: Please include explanation from rebuttal letter for why observer effects are not a concern. In the rebuttal it states ‘we made sure new observer would not differ’ – how, if there were no stats? Worth outlining in supplementary.

REPLY #16: We did not perform any statistics but we clarified the issue in the text, please see the lines 290-296.

L254: Please include explanation from the rebuttal letter.

REPLY #17: Added, see lines 309-313.

L271: See my main comments above about whether colony behaviour is negligible or not.

REPLY #18: Explained in the answer to the main comment, see Reply #3.

L272: I don’t think ‘and their constraints’ adds anything here, and it’s confusing. I suggest removing it.

REPLY #19: Removed

L296: Please include explanation from the rebuttal letter.

REPLY #20: Added, see lines 352-356.

L323-324: ‘Phase’ is used to mean something different for incubation and chick rearing. This is really confusing as they are different metrics (continuous numerical vs categorical) and is misleading. Please rephrase.

REPLY #21: We agree that we are using different metrics for incubation and chick rearing period, but both still represent the timing within the period, so we believe phase is the best way to describe it. Additionally, it would be very inconvenient to say number of days before hatching every time, and we are afraid it would confuse the reader. We explained what we understand under the ‘phase’ term in the lines: 281-285.

L328: What is the distribution of the response variable, please state.

REPLY #22: We clarified this, see lines 389-391.

L360: State that the two chick rearing phases are ‘early’ and ‘mid’ (perhaps in brackets). There is a lot of reliance on readers remembering quite specific pieces of information, please restate these sorts of things when relevant to make it easier.

REPLY #23: Added, line 424.

L364-368: This is still a very difficult sentence to follow and very long. Please rephrase.

REPLY #24: We did our best to rephrase it, see lines 430-436.

L376-377: My confusion here arose because the phrasing suggests that there was a coordination index for both incubation and chick rearing (‘In contrast to the incubation period, we decided to use for the chick rearing period the coordination index’), which I understand from the rebuttal letter is not correct. Please rephrase.

REPLY #25: We removed “in contrast to the incubation” to avoid confusion.

L384: Please include explanation from the rebuttal letter.

REPLY #26: We added more explanation on what phase means to each breeding stage, and why we have two different metrics (see REPLY #21) in the method section presenting recording sessions, and added here when presenting models a reminder to this

L506: I suggest some caveat e.g. behaviour is flexible between breeding stages, because you don’t show intra-individual flexibility and therefore can’t technically refute the ‘sealed bid’ model (e.g. parents could submit a fixed sealed bid for incubation and chick rearing separately, which doesn’t change – this is unlikely but we can’t say with certainty).

REPLY #27: We added some nuance to what we suggest and that more studies are needed to actually disentangle this matter, see lines 583-585.

L544-545: In my original review, I did not mean that hormones don't play a role. My point is that in the discussion, you seem to be asking evolutionary/ultimate questions – i.e. what has driven the evolution of coordinated incubation? For example, you point out that coordination may evolve because it favours embryo development or because it helps to protect chicks prior to thermal independence.

Yes, hormones are likely to be a mechanism by which coordination is achieved – but *why* has this mechanism evolved? It is suggested that hormones explain why birds become more devoted to incubation over time – mechanistically this might be true, but it doesn't answer the 'why' question. Many birds fly south for winter - this could be driven by some hormonally mediated instinct, but that doesn't tell us why they do it in the first place.

It's fine to say that hormonal changes regulate behaviour, but I would put this somewhere else in the discussion and make clear that this is a mechanistic question. In fact, a better option might be to put this in the introduction to help show that levels of coordination change over time. E.g. something along the lines of: prolactin increases over the course of incubation, suggesting changes in parental care behaviour over time - but whether this increases parents’ propensity to incubate, and therefore their level of coordination, is unknown.

REPLY #28: Changes in hormones levels are already present in the introduction to hypothesize their effect on coordination levels. We now added (see line 614 and 622-625) the suggested changes as we agree we have no evidence here on the role of hormones on coordination.

L565-567: Please include explanation from the rebuttal letter.

REPLY #29: Added, see lines 644-649.

L572-573: It seems surprising to expect coordination to ‘reset’ after chick-rearing as outlined in the rebuttal; this needs further explanation (in the manuscript).

REPLY #30: We do not discuss in terms of setting up of coordination anymore, as it is obviously confusing. See lines 654-663.

L582: I think perhaps ‘differences’ would make more sense– instability is still not clear.

REPLY #31: Changed

L594: I suggest ‘reflect’ as ‘express’ suggests an active choice.

REPLY #32: Changed.

L595-596: Please include explanation from the rebuttal letter.

REPLY #33: Added, please see lines 677-685.

Reviewer3

Accept
